# Erythrocyte Membrane Failure by Electromechanical Stress

**DOI:** 10.3390/app8020174

**Published:** 2018-01-25

**Authors:** E Du, Yuhao Qiang, Jia Liu

**Affiliations:** Department of Ocean and Mechanical Engineering, Florida Atlantic University, Boca Raton, FL 33431, USA

**Keywords:** erythrocyte, membrane failure, dielectrophoresis, microfluidics, cell biomechanics, cell lysis

## Abstract

We envision that electrodeformation of biological cells through dielectrophoresis as a new technique to elucidate the mechanistic details underlying membrane failure by electrical and mechanical stresses. Here we demonstrate the full control of cellular uniaxial deformation and tensile recovery in biological cells via amplitude-modified electric field at radio frequency by an interdigitated electrode array in microfluidics. Transient creep and cyclic experiments were performed on individually tracked human erythrocytes. Observations of the viscoelastic-to-viscoplastic deformation behavior and the localized plastic deformations in erythrocyte membranes suggest that electromechanical stress results in irreversible membrane failure. Examples of membrane failure can be separated into different groups according to the loading scenarios: mechanical stiffening, physical damage, morphological transformation from discocyte to echinocyte, and whole cell lysis. These results show that this technique can be potentially utilized to explore membrane failure in erythrocytes affected by other pathophysiological processes.

## 1. Introduction

Mechanical properties of single cells have been proven to be clinically useful biomarkers of various pathological changes, such as cardiovascular disease [1], hematologic diseases [2], and cancer [3]. In erythrocytes, membrane is the only structural component that maintains its mechanical stability when delivering oxygen through the systemic circulation and returning to the pulmonary circulation. During their normal lifespan of 120 days, erythrocytes experience a series of metabolic and physical damages as well as mechanical stresses as they age. Cumulative effects from these factors result in membrane failure, such as vesiculation and mechanical degradation, as well as hemoglobin modification, which transforms a normal discocyte to an echinocyte [4]. Many pathological processes, such as malaria [5], sickle cell anemia [6], hypertension [7], diabetes [8], and such medical procedures as blood banking [9], extracorporeal circuit for hemodialysis [10], and cardiopulmonary bypass [4], can lead to accelerated membrane degradation, remodeling, and failure.

Study of membrane mechanics at the single cell level requires an application of external load to cell membranes and an accurate calibration of the induced stress and strain for individually tracked cells. Many experimental measurement methods, such as micropipette aspiration, atomic force microscopy, microplate stretchers, and optical tweezers, rely on physical contact, the details of which can be found in some review papers [11–13]. Although the physics underlying these techniques can be scaled up, in many existing settings, these techniques suffer from low throughput and can only measure one single cell at a time. To compensate the heterogeneous nature of biological materials, a high-throughput method of loading and measurement on individual cells is required.

**Figure 1:**
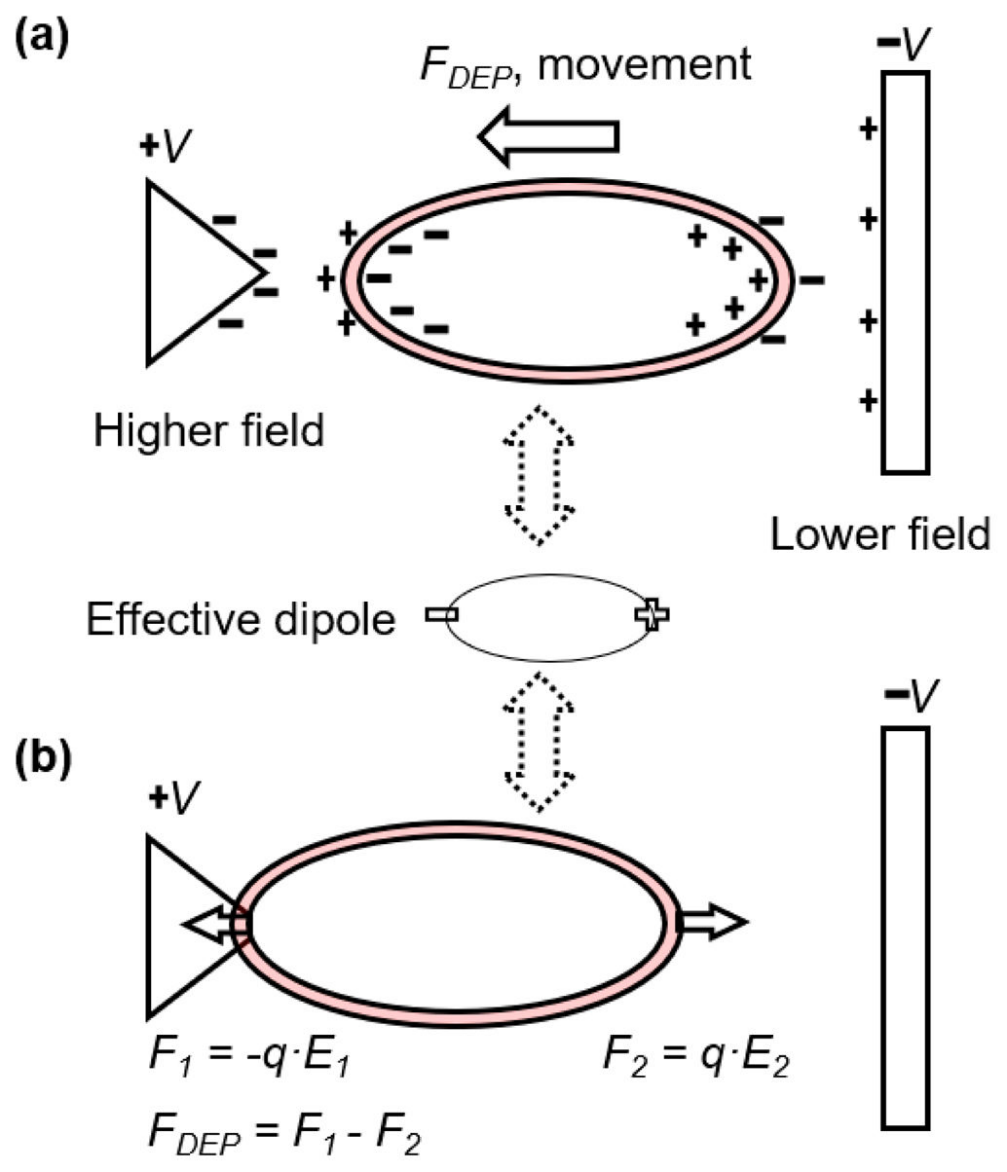
Schematic of electrostatics of biological cells in a non-uniform electric field. (**a**) The interactions between the electric field and the induced dipole charges at a thin ellipsoidal shell generate a net force, which acts to elongate the shell in the direction of electric field. The movement of the shell is known as dielectrophoresis. The direction of cell movement retains when the direction of field changes. (**b**) When the cell approaches the higher field strength and reaches an equilibrium state with no net movement, cell membrane is stretched uniaxially due to the force components in the opposite directions.

Electrodeformation of cell membranes is a relatively new technique that has the potential to measure many single cells at once. When the electric field is non-uniform, the net force exerted on the cell membrane will move the cell toward (or against) the direction of the electric field maxima, given the cell is more (or less) polarizable than the surrounding medium, known as dielectrophoresis (DEP), as shown in Figure 1a. DEP has been widely utilized to separate and sort biological cells in microfluidic settings, the details of which can be found in several review papers [14–18]. Electrodeformation of cell membrane is induced by the electrical forces exerted on the induced charges or by the effective dipole in the cell (Figure 1b). Application of DEP in single cell biomechanics was pioneered by Engelhardt and Sackmann [19], where an inhomogeneous high frequency electric field was used to induce transient electrodeformation of single cells. Such method has been advanced by virtue of microfabrication techniques, such as an interdigitated electrode array, which allows for quantitative measurements of multiple cells at a time. Applications have been found in a wide range of cell types, such as erythrocytes [20–23], platelet [24], mammalian cells [25], plant protoplasts [26], and cervical cancer cells [27].

In this paper, we postulate that the repeated electrical and mechanical stresses can induce membrane changes of single erythrocytes. We bring together the signal modulation technique and DEP to impose cyclic electromechanical loading using mathematical convenience waveforms in a microfluidic setting. This provides an opportunity to test this hypothesis and allows us to explore the progression of membrane failure in circulating erythrocytes, such as mechanical degradation, irreversible membrane damage, and lysis of erythrocytes.

## 2. Materials and Methods

### 2.1. Electrodeformation of Cell Membrane

In a typical biomechanical test where a biological cell is stretched uniaxially, the magnitude of the DEP force on a dielectric ellipsoid particle is expressed by [28]

(1) $$\langle F_{\mathrm{DEP}}\rangle =2\pi \mathrm{abc}\cdot \varepsilon_{m}\cdot Re(f_{CM})\cdot E_{\mathrm{rms}}^{2}$$

where < > is the time-averaged value; *a*, *b*, and *c* are the radii of the particle, *ε_m_* is the permittivity of the surrounding medium, *E_rms_* is the root-mean-square value of the electric field strength, *Re*(*f_CM_*) is the real part of the Clausius–Mossotti factor. The particle will be either attracted to or repelled from a region of high field strength, depending on whether *Re*(*f_CM_*) > 0 or *Re*(*f_CM_*) < 0. To induce electrodeformation of cell membranes by DEP force, a favorable condition is *Re*(*f_CM_*) > 0; the cell moves to a high field strength at electrode edges until an equilibrium condition is reached, characterized by no net movement (Figure 1b). It is noted that other DEP model shall be utilized in the case of spherical particles [28]. The equivalent force F_2_ that is responsible for cellular uniaxial deformation can be calibrated using theories of Maxwell stress tensor [29] and effective dipole moment [25].

Considering a multilayered shell of a biological cell, its effective Clausius–Mossotti factor can be estimated with a single-shell structure, following a concentric multi-shell model [30,31]

(2) $$f_{CM}=\frac{1}{3}\frac{\left(\varepsilon_{\mathrm{mem}}^{*}-\varepsilon_{m}^{*})[\varepsilon_{\mathrm{mem}}^{*}+A_{1}(\varepsilon_{\mathrm{cyto}}^{*}-\varepsilon_{\mathrm{mem}}^{*})]+\rho (\varepsilon_{\mathrm{cyto}}^{*}-\varepsilon_{\mathrm{mem}}^{*})[\varepsilon_{\mathrm{mem}}^{*}-A_{1}(\varepsilon_{\mathrm{mem}}^{*}-\varepsilon_{m}^{*})\right]}{\left(\varepsilon_{m}^{*}+A_{1}(\varepsilon_{\mathrm{mem}}^{*}-\varepsilon_{m}^{*}))[\varepsilon_{\mathrm{mem}}^{*}+A_{1}(\varepsilon_{\mathrm{cyto}}^{*}-\varepsilon_{\mathrm{mem}}^{*})]+\rho A_{2}(1-A_{2})(\varepsilon_{\mathrm{cyto}}^{*}-\varepsilon_{\mathrm{mem}}^{*})(\varepsilon_{\mathrm{mem}}^{*}-\varepsilon_{m}^{*}\right)}$$

where ε^*^ = *ε* − *jσ*/*ω*, $i=\sqrt{-1}$, the subscripts cyto, mem, and m stand for cytoplasm, membrane, and medium, respectively; *ω*, *ε*, and *σ* are the angular frequency, dielectric permittivity, and conductivity, respectively. *ρ* = (*a* − *t*)(*b* − *t*)(*c* − *t*)/(*abc*). Specifically, for a healthy human erythrocyte, literature values [32] suggest *ε_mem_* = 4.44, *ε_cyto_* = 59, *σ_mem_* = 10^−6^ *S*/*m*, and *σ_cyto_* = 0.31 *S*/*m. A_i_*=_1,2_ is the depolarization factor:

(3) $$A_{i}=\frac{a_{i}b_{i}c_{i}}{2}\int_{0}^{\infty }\frac{d_{s}}{\left(s+a_{i}^{2}\right)\;B_{i}},\;i=1,2$$

where $B_{i}=\sqrt{\left(\left(s+a_{i}^{2}\right)\;\left(s+b_{i}^{2}\right)\;\left(s+c_{i}^{2}\right)\right)}$, *a*_1_ = *a*, *b*_1_ = *b*, *c*_1_ = *c*, *a*_2_ = *a* − *t*, *b*_2_ = *b* − *t*, *c*_2_ = *c* − *t*, and *t* is the thickness of the erythrocyte membrane (4.5 nm).

### 2.2. Sample Preparation

The erythrocyte samples were prepared from the whole blood collected from a healthy donor. Plasma, platelets, and leukocytes were removed by washing the blood with phosphate-buffered saline buffer (PBS, Lonza Walkersville, Inc., Walkersville, MD, USA) twice at room temperature. The collected erythrocytes were diluted into an isotonic working buffer [33], which contained 8.5% (*w*/*v*) sucrose and 0.3% (*w*/*v*) dextrose with the electrical conductivity adjusted to 0.018 S/m using PBS for favorable operation of erythrocyte suspension. The final concentration of erythrocytes was 10^6^ cells/mL for all electromechanical deformation testing.

### 2.3. Experimental Electrodeformation Tests

We constructed a microfluidic platform to investigate the electrodeformation-induced membrane failure in biological cells. The platform consisted of a polydimethylsiloxane (PDMS) microchannel 50 μm in depth and a glass substrate coated with a micropatterned titanium/gold (10/100 nm thick) electrode array, following a protocol presented in previous work [22]. The interdigitated electrode fingers had a 20 μm gap and a 20 μm band width, generating a sufficient field strength using low voltages supplied by a signal generator. Radio-frequency sinusoidal waveform modified with an amplitude-shift keying technique was supplied to the electrodes. Before loading the sample, the microfluidic channel was primed with a working buffer containing 5% bovine serum albumin for 30 min to prevent cell adhesion.

**Figure 2:**
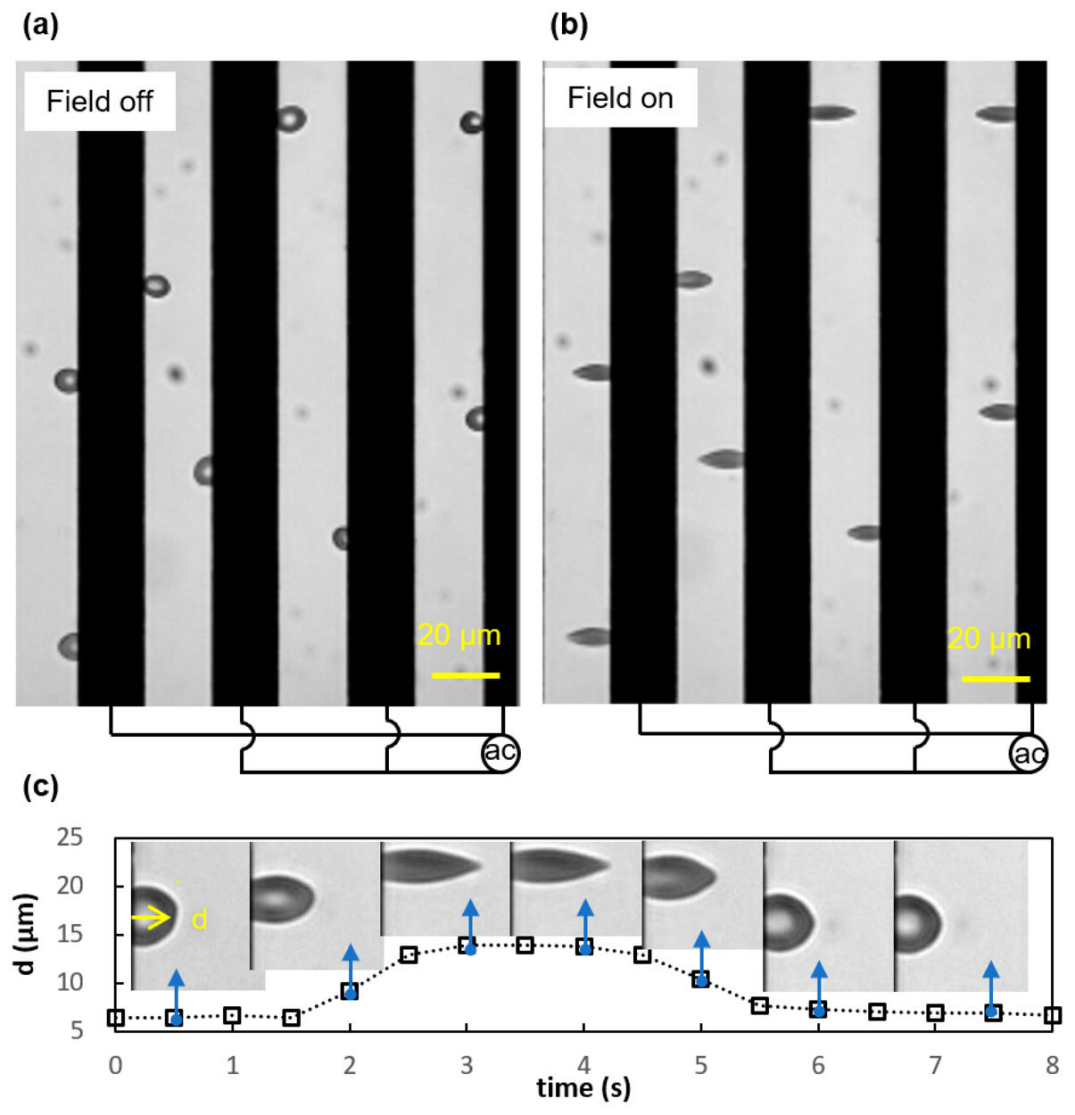
Experimental observations of electrodeformation of erythrocyte membranes in interdigitated electrode settings. (**a**) Cells are freely suspended in the medium when the electric field is off. (**b**) Cells are stretched by the DEP force when the electric field is supplied. (**c**) Representative electrodeformation of the cell membrane, *d*, as quantified by the maximum displacement of the cell membrane to the edge of the electrode. Insets show representative deformation of the cell membranes with time.

Electrodeformation of erythrocyte membranes was carried out by subjecting the erythrocyte suspension to an alternating current electric field (Figure 2). A series of electrodeformation tests of the erythrocyte membranes were performed. In the viscoelastic testing, an erythrocyte membrane was subjected to a small electrodeformation. The cell membrane could return to its original shape after the external electric field was removed. In the plastic deformation, an erythrocyte would not return to its original shape after the external load was removed. To achieve membrane failure, two experiments were performed: (i) A dynamic experiment, where the electromechanical stress varied cyclically in the mathematical convenience waveforms with time, i.e., square and sinusoid, was conducted, and the response of the cell was measured at loading frequencies of 0.1 Hz and 0.25 Hz, respectively. The transient creep and recovery processes were analyzed, and the plastic deformation was noted. (ii) An erythrocyte membrane was subjected to a high voltage excitation at 7 *V_rms_* for a long period of time (>20 s); this long pulse of electromechanical stress caused great electrodeformation, where the ratio between the major and minor axes was greater than 1.5 [34]. Erythrocyte deformation was visualized by an Olympus X81 inverted microscope and recorded for quantitative analysis. To enable the above testing conditions, the electric frequency of the carrier sinusoidal waveform was set at 1.58 MHz, which allowed us to generate a favorable tensile stretching condition with minimum voltage levels in current settings. Its magnitude was modulated by a square waveform and a sinusoidal waveform at different frequencies using the amplitude shift keying technique.

## 3. Results and Discussion

### 3.1. Viscoelastic Behavior and Fatigue Failure

**Figure 3:**
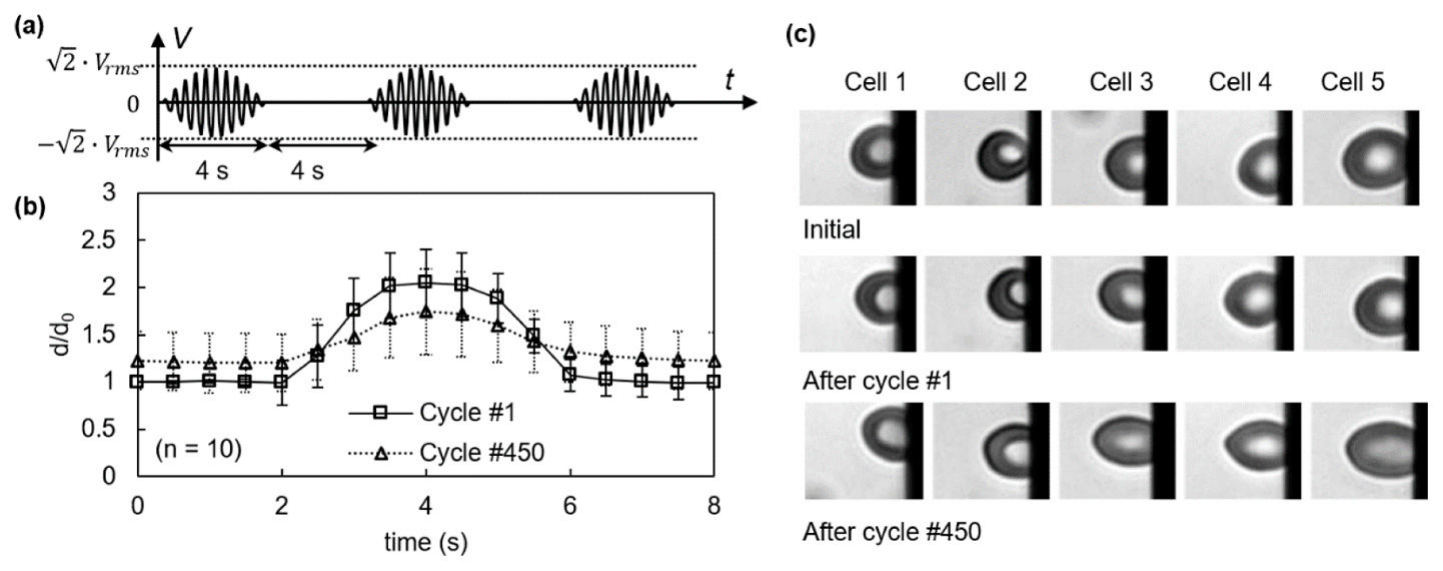
Electrodeformation of erythrocyte membranes during cyclic sinusoidal waveforms. (**a**) Electrical excitation was supplied to the erythrocyte suspension, each cycle consists of a duration for 4 s and a relaxation for 4 s. *V_rms_* value is 2 V. (**b**) Displacement of erythrocyte membrane to the electrode edge as a function of time (*n* = 10) during the 1st and 450th cycles, respectively. Error bar represents the standard deviation. (**c**) Morphological of cells before electrodeformation and after the 1st and 450th cycles of electrodeformation.

**Figure 4:**
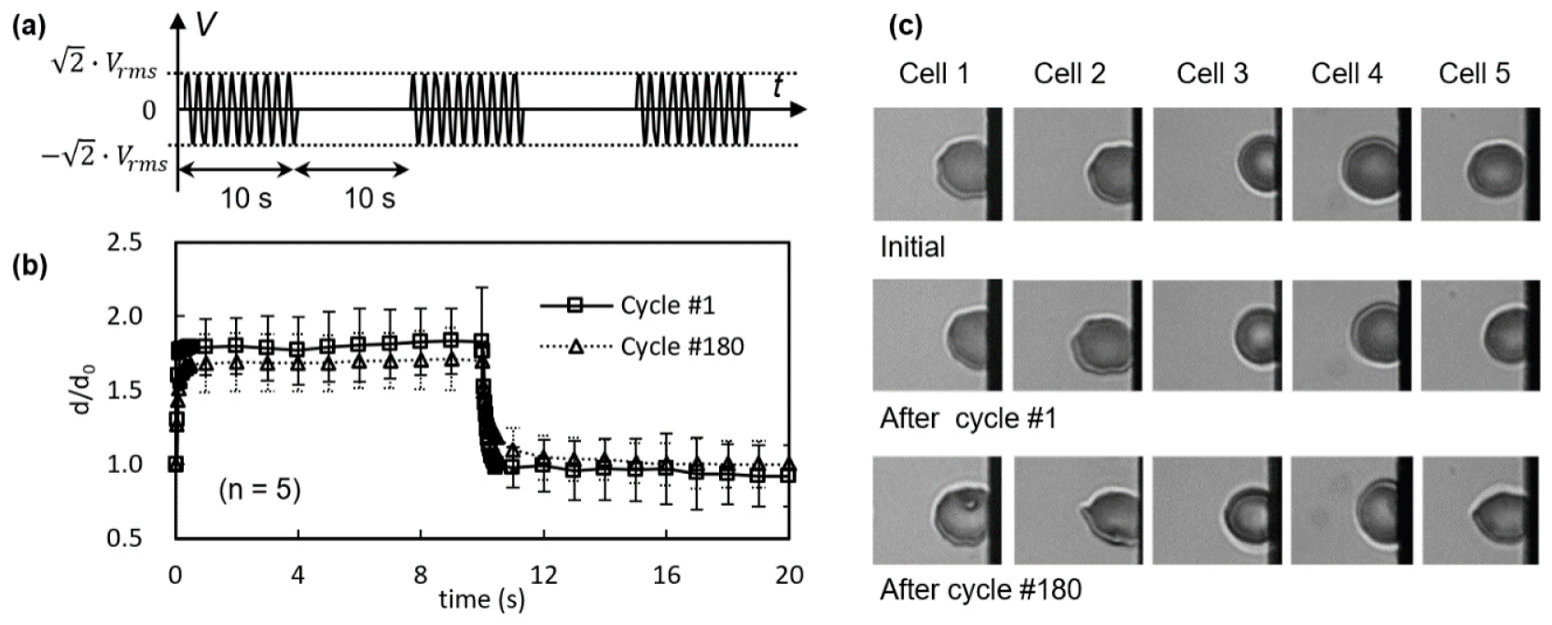
Electrodeformation of erythrocyte membranes during cyclic square waveforms. (**a**) Electrical excitation was supplied to the erythrocyte suspension, each cycle consists of a duration for 10 s and a relaxation for 10 s. *V_rms_* value is 2 V. (**b**) Displacement of erythrocyte membrane to the electrode edge as a function of time (*n* = 5) during the 1st and 180th cycles, respectively. Error bar represents the standard deviation. (**c**) Morphological of cells before electrodeformation and after the 1st and 180th cycles of electrodeformation.

Viscoelastic testing was carried out by subjecting the erythrocyte suspension to a short pulse of electrical excitation at 2 *V_rms_* and dynamic testing was conducted using the cyclic pulses of above electrical excitations. A sinusoidal wave pulse for 4 s (Figure 3a) and a square wave pulse for 10 s (Figure 4a) were utilized for both experiments. The maximum value of the carrier sinusoidal waveform was 2.8 kV/m, which was much lower than the critical field strength—20–40 kV/m is needed for breakdown of the plasma membrane [35,36]. Deformation of the cell was quantified as the maximum displacement of cell membrane to the edge of the electrode relative to the initial displacement when the cell started to respond to the electric field, *d*/*d*_0_.

Erythrocytes exhibited time-dependent viscoelastic behavior (Figures 3b and 4b). During the 1st application of electromechanical stress to a sinusoidal waveform excitation, cell membranes deformed gradually, reached their maximum state of 2.05 ± 0.34, and recovered gradually upon the unloading process, as determined from 10 different measurements. Upon a gradual release of the loading, erythrocyte membranes recovered gradually and eventually returned to their initial state with no noticeable plastic deformation (Figure 3c). During the 450th cycle, the maximum deformation of erythrocyte membranes was reduced to 1.74 ± 0.46. Cell membranes responded to the loading and unloading much slower than the initial cycles. After the 450 cycles of loading, they could not return to their initial states after long relaxation. Erythrocytes turned into ellipsoidal shapes (Figure 3c, Cells 2–5).

During the 1st cycle of square waveform excitation, cell membranes responded instantly and reached to their maximum deformation level, 1.83 ± 0.23, determined by five different measurements (Figure 3). Upon a sudden release of loading, erythrocyte membranes recovered quickly and returned to their initial state with no noticeable plastic deformation. After 180 cycles of excitation, the maximum deformation level was reduced to 1.71 ± 0.20, and quick recovery was observed. However, permanent damage was formed in the cell membranes, in the form of thorny projections at the far edge of erythrocyte membranes (Figure 4c, Cells 2 and 5). In another study, reciprocated mechanical stresses were achieved by forcing cells to squeeze through a narrow microfluidic channel [37]. It was found that changes in cell shapes, evaluated by cell length and deformation index, were related to the number of cell passage cycles. Due to the different loading conditions, cell membranes exhibited different morphologies after cyclic stresses. In the electrodeformation method, the effective electrical forces were primarily exerted on the two poles of a cell. The cyclic forces were likely distributed at the same locations. In contrast, in the shear flow method, the shear stresses were distributed across the cell membranes. It should be noted that, in the current study, erythrocytes were suspended in an isotonic buffer. Altering the extracellular characteristics may lead to different electromechanical measurements, as erythrocyte deformation is strongly dependent on osmotic pressure, determined by the ektacytometry technique [38].

**Figure 5:**
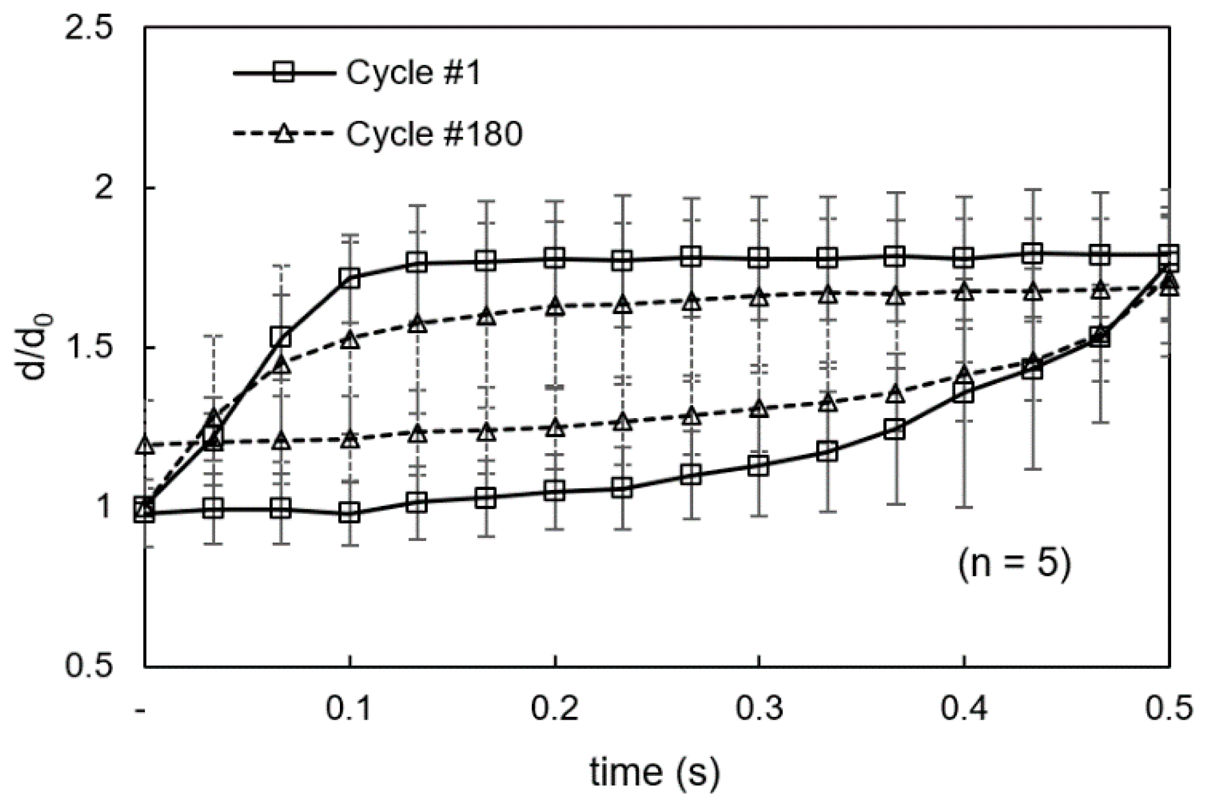
Creep and recovery test during cyclic loading conditions. Error bar represents the standard deviation.

The details of the membrane’s creep-recovery behavior were obtained by observing the cellular response to the above square waveform loading condition. This behavior is similar to that of a typically encountered viscoelastic material to a certain extent. Erythrocyte membrane responds with a strain gradually increasing with time and the gradual recovery starts upon release of the load (Figure 5). It is interesting that, during the 1st cycle, at such a constant loading, the recovery process of the erythrocyte membrane was eventually completed and was similar to a viscoelastic solid. After 180 cyclic stresses, both the deformation rate and the maximum deformation of the erythrocyte membranes were apparently lower than they were in the initial cycle, indicating an increase in membrane viscosity and shear elasticity from fatigue. After the electromechanical stress was removed, a residue of deformation was left in cell membranes.

### 3.2. Membrane Failure Due to High Field Strength

**Figure 6:**
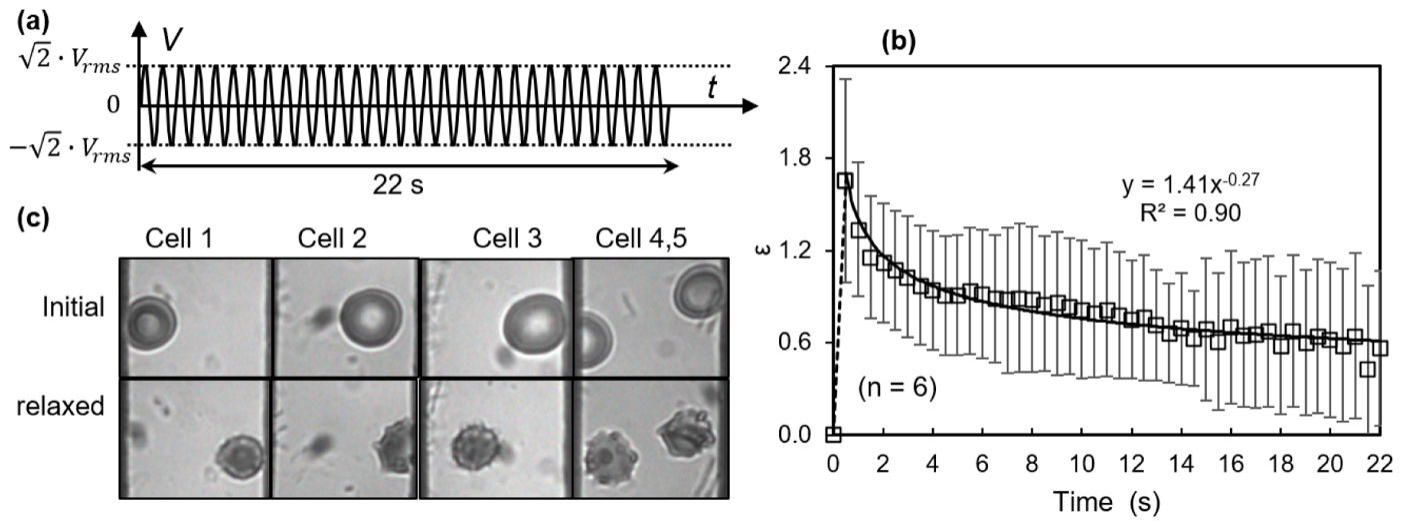
Erythrocyte response to high field strength excitations. (**a**) Induced membrane failure test was conducted by subjecting erythrocyte suspension to a constant high electric field strength for a duration of 22 s and removed after that. *V_rms_* value is 7 V. (**b**) Such a load caused a strain in erythrocyte membranes as a function of time. It decreased gradually in an exponential function (*n* = 6). Error bar represents the standard deviation. (**c**) After the loading condition was removed, cells recovered with permanent damages in the membranes, similar to the echinocytes with small volume and thorny projections at multiple places.

Membrane failure was also observed when subjecting the erythrocyte suspension to a higher voltage excitation, 7 *V_rms_* (Figure 6a). Mechanical strain, *ε* = (*d* − *d*_0_)/*d*_0_ was averaged from six different measurements. The value of *ε* increased within the first second and gradually decreased in an exponential function within the duration of loading. After the electrical field was removed, erythrocytes recovered but never returned to their initial states (Figure 6c). Eventually, erythrocytes membranes showed multiple thorny projections similar to a typical echinocyte. The dimple area disappeared, and an apparent reduction in cell volume was observed. This failure mode was different from the membrane damage observed in the fatigue test, where only one thorny projection was formed in the fraction of cells under the same type of square waveforms.

Another interesting observation was that a small number of erythrocytes under 7 *V_rms_* excitations turned into ghost cells, indicating the release of hemoglobin from erythrocytes. Figure 5a shows the time lapse of a representative cell under electrodeformation for 4.5 s. The cell responded to the electric excitation within 0.5 s and was fully stretched, retracted rapidly, and gradually became transparent, observed under the bright-field microscopy through a band pass filter of 414 ± 46 nm, which is near the peak of the hemoglobin absorption spectra. The field pulse was long enough; it is likely that the electrical breakdown of erythrocyte membranes occurred at such a high electric field strength, where the maximum value of the carrier sinusoidal waveform is up to 5 kV/m. Although the field strength utilized here was below the critical field strength required for the breakdown of membranes, 20–40 kV/m [35,36], it is likely that the electromechanical coupling exerted high tensile stress that contributed to the lysis of cellular plasma membranes and opened up pathways for hemoglobin release.

**Figure 7:**
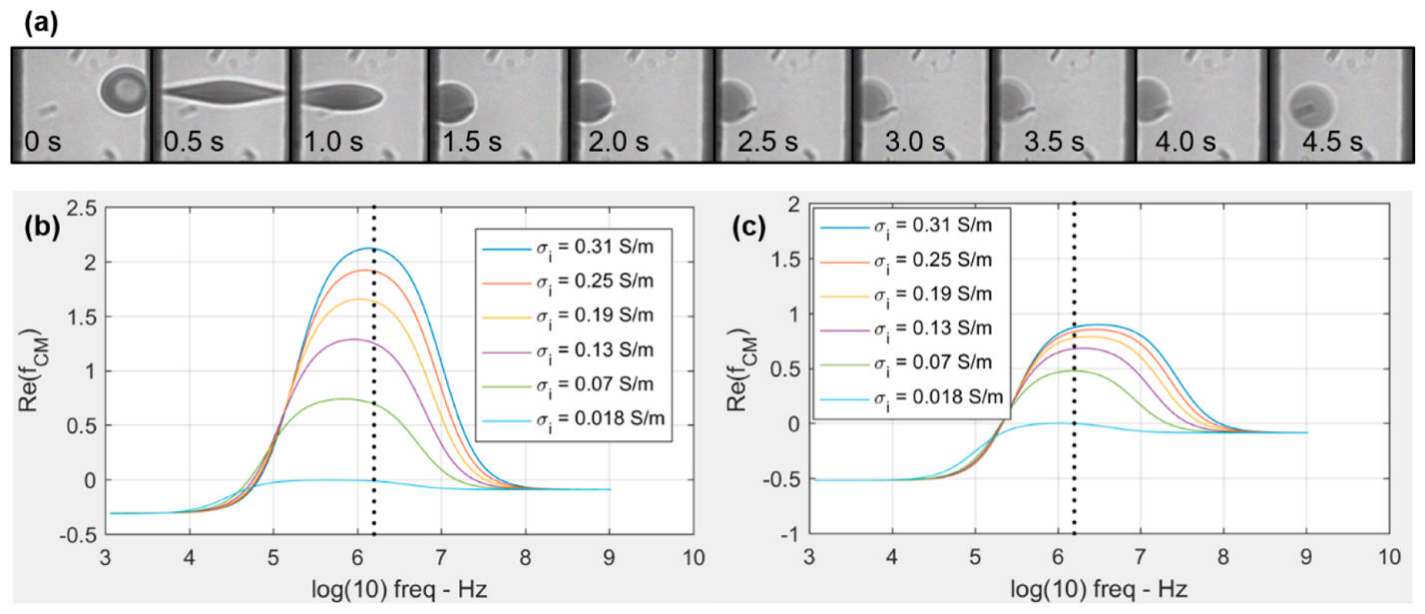
Release of hemoglobin from erythrocytes upon the coupling of membrane electrical breakdown and the electromechanical tensile stress. (**a**) Time elapse of a representative erythrocyte in response to 7 *V_rms_* excitation. (**b**, **c**) *Re*(*f_CM_*) as a function of electrical frequency for erythrocytes with different interior electrical conductivity, using ellipsoid and spherical models of the same volume, respectively. The three principal axes of the ellipsoidal erythrocyte were assumed to be 5 μm, 1.5 μm, and 1.3 μm, respectively. The dashed line indicates the excitation frequency of 1.58 MHz used in this study.

The effects of the induced release of hemoglobin were also recognized from the smaller deformation of erythrocyte membranes under a constant high electric field strength. As the electrical conductivity of erythrocyte interior is a function of hemoglobin concentration, its electrical conductivity is expected to decrease with the loss of hemoglobin. This process affects the Clausius–Mossotti factor, *Re*(*f_CM_*), which is proportional to the magnitude of the DEP force exerted on cell membranes (Equation (1)). The value of *Re*(*f_CM_*) decreases from 2.1 to 0.7 according to the ellipsoid model, and 0.87 to 0.48 according to the spherical model [21], when the interior conductivity of erythrocyte reduces from 0.31 to 0.07 S/m (Figure 7b, c). Correspondingly, when the interior conductivity of erythrocyte reduces to a same level as the surrounding medium, 0.018 S/m, the value of *Re*(*f_CM_*) further reduces to −0.015 and −0.004 based on the ellipsoidal and spherical models, respectively, both indicating a negative DEP. These mathematical predictions were confirmed by experimental observations in that the transparent ghost erythrocytes were slowly repelled away from the higher field strength.

## 4. Conclusions

We have presented a new application of electrodeformation technique for the study of membrane failure in normal erythrocytes. We confirmed that the cyclic electromechanical stress can induce irreversible membrane failure, including the mechanical degradation and permanent damage in cell membranes, using short pulses of electric excitations that were about 10% of the required field strength for membrane breakdown. Another mode of membrane failure, the discocyte–echinocyte transformation was induced by a field strength that was higher but still below the critical membrane breakdown strength. Additionally, we observed that a small fraction of cells turned into ghost cells, indicating hemoglobin escape and confirmed with the alternated DEP behavior. These results indicate that the coupling between the electrical stress and mechanical deformation both contribute to membrane failure in human erythrocytes. We envision that this method will provide new opportunities for studying membrane failure in erythrocytes affected by aging and diseases.
